# A Real-Time Monitoring Method for Droplet Transfer Frequency in Wire-Filled GTAW Based on Arc Sensing

**DOI:** 10.3390/s24061924

**Published:** 2024-03-17

**Authors:** Aiting Jia, Yifang Luo, Bo Hong, Xiangwen Li, Li Yin, Mina Luo

**Affiliations:** 1 Schoool of Mechanical Engineering and Mechanics, Xiangtan University, Xiangtan 411105, China; jat0929@163.com (A.J.); lyfang99999@163.com (Y.L.); hongbo@xtu.edu.cn (B.H.); 16670798661@163.com (M.L.); 2 Engineering Research Center of Complex Track Processing Technology & Equipment, Ministry of Education, Xiangtan University, Xiangtan 411105, China

**Keywords:** GTAW, arc sensing, droplet transfer, uniformity of droplet transition

## Abstract

Droplet transfer frequency is a decisive factor in welding quality and efficiency in gas tungsten arc welding (GTAW). However, there still needs to be a monitoring method for droplet transfer frequency with high precision and good real-time performance. Therefore, a real-time monitoring method for droplet transfer frequency in wire-filled GTAW using arc sensing is proposed in this paper. An arc signal acquisition system is developed, and the wavelet filtering method filters out noise from the arc signal. An arc signal segmentation method—based on the OTSU algorithm and a feature extraction method for droplet transition based on density-based spatial clustering of applications with noise (DBSCAN)—is proposed to extract the feature signal of the droplet transition. A new conception of droplet transition uniformity is proposed, and it can be used to monitor the weld bead width uniformity. Numerous experiments for monitoring droplet transfer frequency in real time are conducted with typical welding parameters. This method enables the real-time observation of droplet transfer frequency, and the result shows that the average monitoring error is less than 0.05 Hz.

## 1. Introduction

Gas tungsten arc welding (GTAW), known for its advantages such as reduced spatter, a highly stable arc, high quality, and ease of automation [1,2,3], is widely used in additive manufacturing and surface repair [4,5]. Droplet transfer is critical in determining the weld quality [6]. For high-quality welding beads and high efficiency, it is necessary to make the metal droplet transfer stable and controllable during welding. So droplet transfer of high stability is indispensable, and the stability will be affected by gravity, surface tension, self-induced electromagnetic force (SEM), the wire-filled position, and other factors [7]. Because of these influences, the stability of droplet transfer is difficult to ensure, which seriously affects the welding quality. A reliable approach to maintaining the stability of droplet transfer in wire-filled GTAW is closed-loop control [8], the key to which involves monitoring the droplet transfer frequency in real time [9,10].

Nowadays, many studies focus on reducing droplet size [11,12], controlling droplet transfer modes [13], accelerating the droplet transition frequency [14,15,16], and so on, to monitor and control the stability of droplet transfer. Wu et al. [17] recorded the metal transfer process in double-wire pulsed GMAW using a high-speed camera and established a mathematical model of the droplet diameter with different arc waveforms; this model more accurately describes changes in the output current during the actual welding process and the influence of welding parameters on droplet size. Jorge et al. [18] analyzed the size of the droplets and transfer frequency by observing the fluctuation on the surface of the melt pool using a high-speed camera system. Chang et al. [19] proposed a real-time monitoring method based on machine vision for droplet transfer distance and achieved a stable liquid bridge process by adjusting the height of the base in real time. Solano et al. [20] developed an algorithm that can recognize droplets accurately and they achieved the recognition of droplet shapes and the extraction of droplet sizes from photos captured by a high-speed camera. Wang et al. [21] developed a laser back-lighting-based monitoring system to obtain photos of the droplet transfer process and proposed a double-threshold method to segment the image robustly so that they could extract the droplet transfer information smoothly. Pérez et al. [22] proposed a segmentation model based on a deep learning architecture using FCNs to extract information about the droplet transfer process from photos obtained by a high-speed camera system; this approach is faster and more accurate. Chaurasia et al. [23] monitored the droplet transfer process using a high-speed camera in real time and proved that it is viable to develop a closed-loop control system based on the dynamic droplet transfer process to improve the stability of welding. Teixeira et al. [24] found the range of arc voltages and currents corresponding to different droplet transfer modes, enabling control over these droplet transfer modes. These studies realize the real-time observation of the droplet transfer process through direct visual monitoring methodologies and facilitate the extraction of information about this process using image processing algorithms. Still, there are obvious disadvantages to visual monitoring methodology. It is economically inefficient to monitor the droplet transfer process with visual monitoring methodology because of spatter, fog, intense arc light, and other complex disruptions that are hard to avoid during welding; moreover, the large amount of data involved in image processing challenges the capability of existing algorithms to fulfill the requirements of real-time monitoring and feedback control [25].

Instead, with simple construction and no need for expensive equipment, arc sensors are economical [26,27]. During GTAW, the arc voltage value corresponds to the arc length unaffected by other complex disturbances, like arc light, spatter, and fog, so that the change rule of the arc length can be reflected by that of the arc voltage value with great performance stability and in real time [28]. So arc sensors are widely used for real-time monitoring and control of the welding process because of these characteristics [29,30,31,32]. Sergio Ríos et al. [33] believed that the arc voltage drops due to the contact between the wire and the pool through the droplet, which changes the pool form and shortens the average arc length; once the wire and pool lose touch with each other, the arc voltage suddenly restores to its original state; the authors pointed out that this discovery can be used in monitoring droplet transfer processing [34], but they have not proposed a certain monitoring method. Qin et al. [35] processed arc signals using deep learning based on LSTM-NN (long short-term memory neural network), according to the change rule of arc signals across different droplet transfer modes in WAAM (wire arc additive manufacturing); this approach successfully classifies and controls the droplet transfer mode, improving arc stability. However, there is still no in-depth research on the real-time monitoring of droplet transfer frequency.

To monitor droplet transfer frequency in closed-loop feedback control of wire-filled GTAW, in this paper, we propose a real-time monitoring method for droplet transfer frequency in wire-filled GTAW using arc sensing. Firstly a real-time monitoring system for droplet transfer frequency is established; second, we obtain arc signals in real time during welding and filter out noise from original arc signals using the proposed filtering method based on wavelet transform; we then segment the filtered signals into the droplet growth signal and droplet transfer signal using the proposed arc signal segmentation method based on OTSU. Finally, we obtain the feature signal of the droplet transition using the proposed feature extraction method for droplet transition based on DBSCAN, and we calculate the droplet transfer frequency and the uniformity of the droplet transition.

## 2. Principles

**Figure 1:**
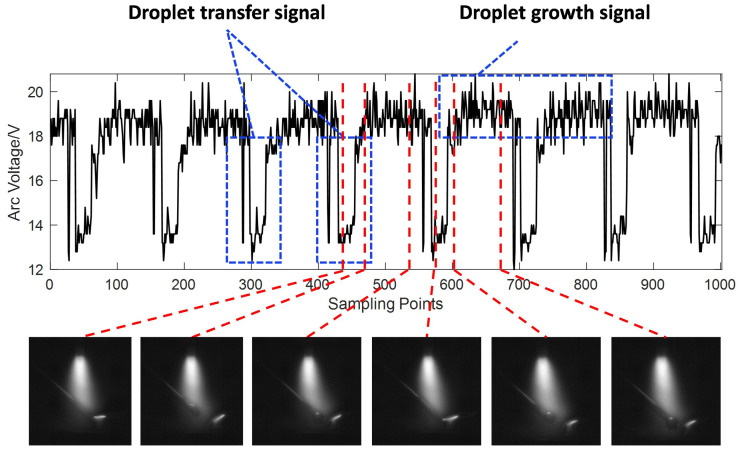
The original arc voltage signal.

Figure 1 shows the original arc voltage during wire-filled GTAW. As shown in Figure 1, with the droplet growing up, the wire will touch the welding pool through the droplet and shorten the average arc length, leading to arc voltage descending sharply. When the droplet finally detaches from the wire, the wire separates from the welding pool. So, the arc length will be restored to its original size, and the arc voltage will increase accordingly (this kind of arc signal is called a droplet transfer signal). After that, a new droplet will form at the tip of the wire, and grow for a while. During growth, the arc voltage will remain stable and high (this kind of arc signal is called a droplet growth signal). Based on this principle, the droplet transfer information is available by processing the arc signals. In this paper, we propose a real-time monitoring method for the droplet transfer frequency in wire-filled GTAW using arc sensing. This method enables the real-time observation of droplet transfer frequency by processing the arc voltage data during wire-filled GTAW.

**Figure 2:**
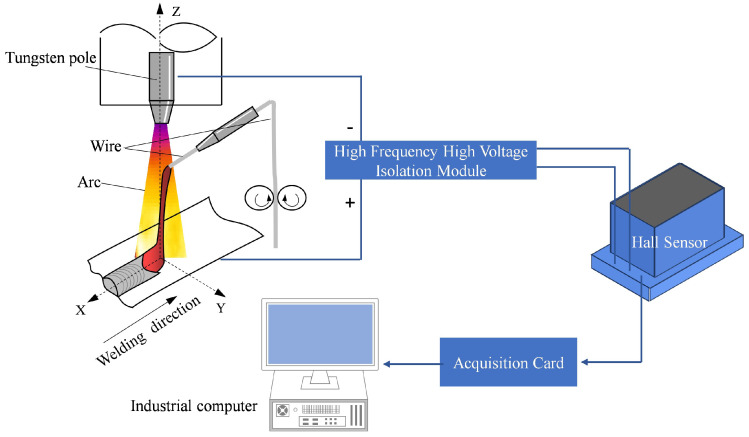
System for the real-time monitoring method for droplet transfer frequency in wire-filled GTAW using arc sensing.

**Figure 3:**
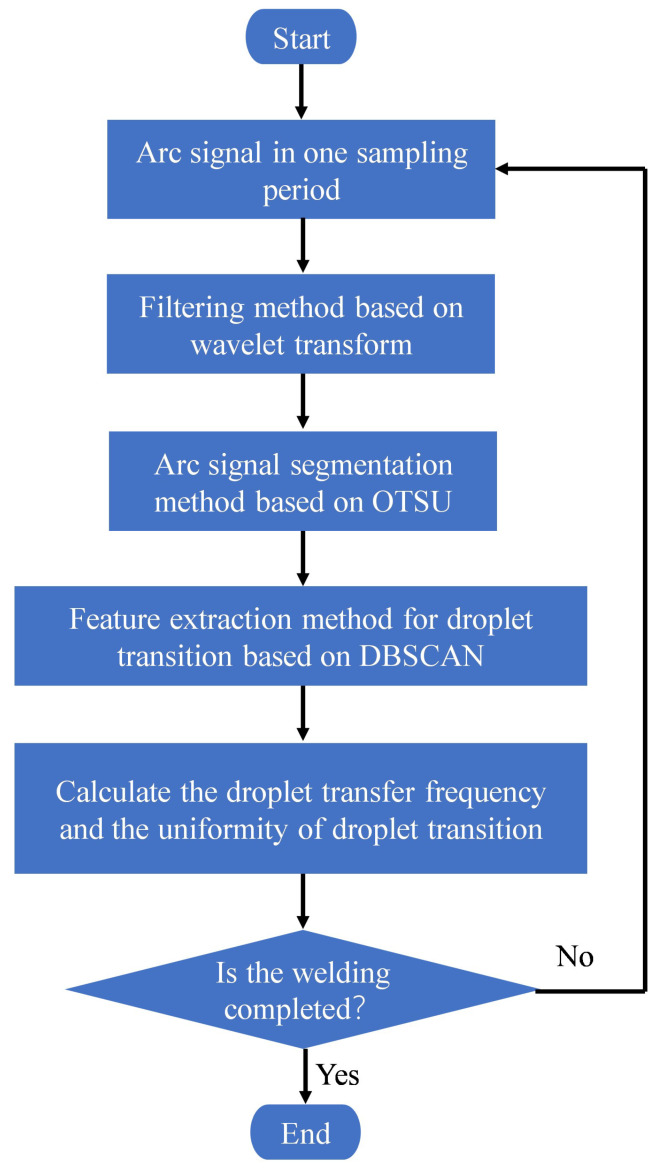
The flow chart of the real-time monitoring method.

Figure 2 presents a schematic diagram of the real-time monitoring method for droplet transfer frequency in wire-filled GTAW using arc sensing. During GTAW, arc signals are obtained by a hall sensor and processed by an industrial computer in real time using the real-time monitoring method for droplet transfer frequency in wire-filled GTAW using arc sensing, enabling the real-time observation of droplet transfer frequency. The flow chart of the proposed method is shown in Figure 3.

To ensure the accuracy of this method, the arc signal is filtered using the wavelet filtering method to reduce noise interference. Then, an arc signal segmentation method based on OTSU is proposed, which can precisely segment the arc signal into the droplet growth signal and droplet transfer signal. Moreover, a feature extraction method for droplet transition based on DBSCAN is proposed to precisely extract the feature signal of the droplet transition so that the droplet transfer frequency and the uniformity of the droplet transition can be calculated.

### 2.1. Filtering Method Based on Wavelet Transforms

The original arc voltage signal is shown in Figure 1. There is noise in the original arc voltage signal since there is electromagnetic interference and an arc self-loading state during welding, which will affect the accuracy of feature extraction. To improve the extraction accuracy, it is necessary to filter out the noise. The original arc voltage signal consists of a series of discrete time series, and the arc voltage signal model is shown in Equation (1):

(1) $$U\left(\tau_{n}\right)=V_{c}\left(\tau_{n}\right)+V_{g}\left(\tau_{n}\right)+e\left(\tau_{n}\right)$$

where *n* is the *n*th sampling point; $\left\{\tau_{n}\right\}$ is the time series corresponding to the sampling point; $U\left(\tau_{n}\right)$ is the original arc voltage signal; $V_{c}\left(\tau_{n}\right)$ is the droplet growth signal; $V_{g}\left(\tau_{n}\right)$ is the droplet transfer signal; and $e(\tau_{n})$ is the noise signal.

**Figure 4:**
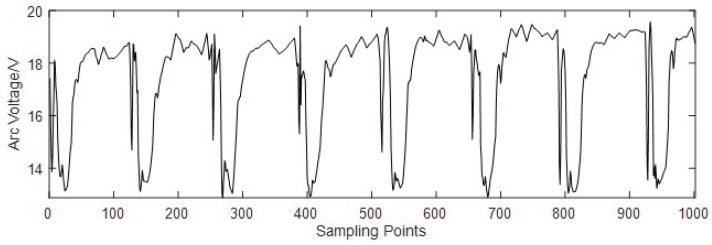
The filtered arc voltage signal.

As shown in Figure 1, the data that we process have large local changes. In existing methods, the wavelet transform is more appropriate than others for data with large local changes. The wavelet transform is characterized by its multi-scale and multi-resolution capabilities, offering unparalleled advantages over traditional methods regarding non-smooth signal denoising and feature extraction. So, we propose a filtering method based on wavelet transform to process the original arc voltage signal. After filtering, the relatively smooth arc voltage signal without noise is shown in Figure 4. The filtered arc voltage signal retains the changing trends of the original one without too much high-frequency noise. In addition, the level and width of the high-level voltage and low-level voltage remain the same. So, the filtered arc voltage signal meets the requirements of extracting the feature signals of the droplet transition.

### 2.2. Arc Signal Segmentation Method Based on the OTSU Algorithm

**Figure 5:**
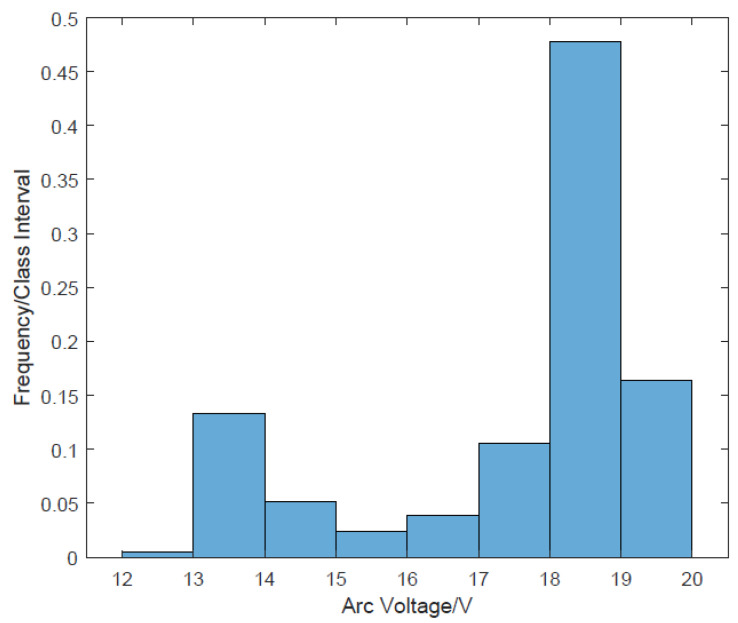
The frequency histogram of the arc signal.

As shown in Figure 4, the droplet growth signals are high-level, and the droplet transfer signal points are low-level. Figure 5 presents a frequency histogram, showing the frequencies of occurrence of different signal point values. The frequency obeys the bimodal distribution.

OTSU (the maximum inter-class variance method, an algorithm for image binarization proposed by OTSU) [36] is suitable for the binarization threshold selection of grayscale images with overall “bimodal” characteristics in image grayscale distribution. Based on the principle of OTSU, we propose an arc signal segmentation method based on OTSU to segment the droplet growth signal and the droplet transfer signal. The steps are as follows:

First, we arrange the arc signal values in descending order. If there is a total of *k* arc voltage values, we count the number of occurrences of each arc signal value. $n_{i}$ represents the occurrences of the *i*th value, where i=1,2,3,......,k.

Second, we calculate the frequency of every value with Equation (2):

(2) $$\begin{array}{c} f_{i}=n_{i}/N \end{array}$$

(3) $$\begin{array}{c} N=\sum_{1}^{k}n_{i} \end{array}$$

where $f_{i}$ is the frequency of every value, *N* is the arc signal data number per sampling period.

Third, we calculate the between-class variance with Equation (4):

(4) $$\begin{array}{c} G_{i}=\omega_{0i}\times \omega 1i\times {\left(\alpha_{0i}-\alpha_{1i}\right)}^{2} \end{array}$$

(5) $$\begin{array}{c} \omega_{0i}=\sum_{1}^{i}f_{i} \end{array}$$

(6) $$\begin{array}{c} \omega_{1i}=\sum_{i}^{k}f_{i} \end{array}$$

(7) $$\begin{array}{c} \alpha_{0i}=\sum_{1}^{i}f_{i}\times i \end{array}$$

(8) $$\begin{array}{c} \alpha_{1i}=\sum_{i}^{i}f_{i}\times i \end{array}$$

where $G_{i}$ is the between-class variance; when assuming that the threshold is the *i*th value, $\omega_{0i}$ is the sum of the frequencies of the first *i* arc voltage values, $\omega_{1i}$ is the sum of the frequencies from the *i*th value to the last, $\alpha_{0i}$ is the average of the first *i* arc voltage values, and $\alpha_{1i}$ is the average from the *i*th value to the last.

Fourth, we calculate the maximal between-class variance and obtain the threshold with Equations (9) and (10):

(9) $$\begin{array}{c} G_{max}=\underset{1\leq i\leq k}{max}\;G_{i}=G_{j} \end{array}$$

(10) $$\begin{array}{c} V_{th}=V_{j} \end{array}$$

where $G_{max}$ is the maximal between-class variance, and $V_{th}$ is the threshold.

**Figure 6:**
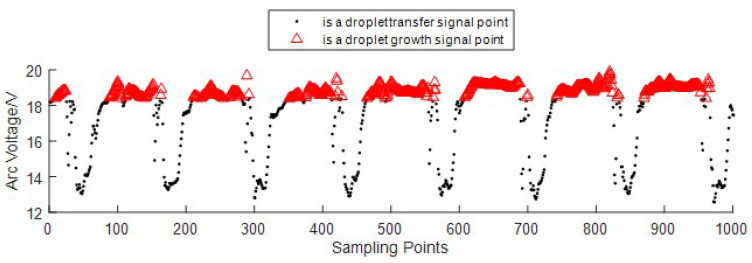
The segmentation result.

The segment result is shown in Figure 6. The droplet growth signal and droplet transfer signal are in different collections, $V_{g}$ and *D*.

(11) $$\begin{array}{c} V_{g}=\left\{x_{g}|x_{i}\geq V_{th}\right\} \end{array}$$

(12) $$\begin{array}{c} D=\{x_{c}|x_{i}\in V,x_{i}\notin V_{g}\} \end{array}$$

where $V_{g}$ denotes the collection of droplet growth signals, $x_{i}$ denotes the *i*th value, $V_{th}$ denotes the threshold, *V* denotes the collection of all the signals in one sampling period, and *D* denotes the collection of droplet transfer signals.

### 2.3. Feature Extraction Method for Droplet Transition Based on DBSCAN

**Figure 7:**
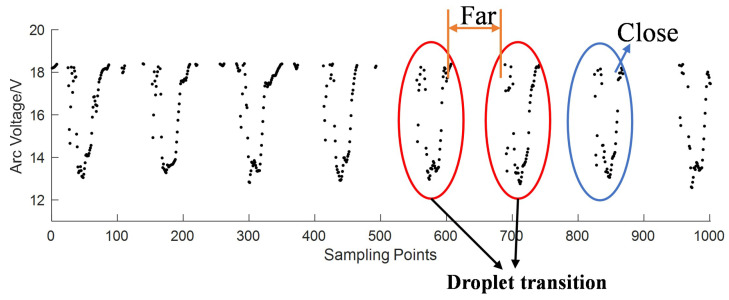
The remaining droplet transfer signals, *D*.

After segmentation, the droplet transfer signals, *D*, as shown in Figure 7, are retained to extract the feature signals of the droplet transition.

**Figure 8:**
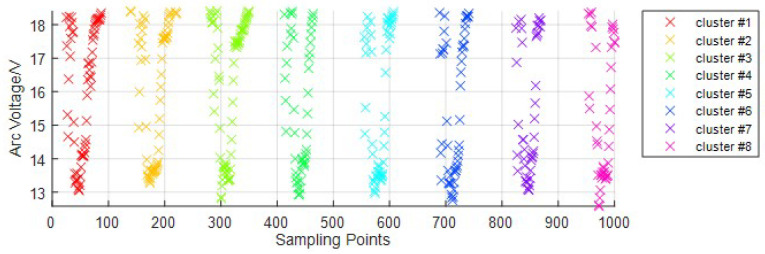
The extraction result of the droplet transition.

In wire-filled GTAW, the arc voltage will be at a low level when the droplet contacts the pool, and at a high level when the droplet grows. In every sampling period during welding, there will be some droplets. Every time one droplet transfers into the welding pool, we call it one droplet transition. Every droplet transition has its own arc voltage signal points, which are close to each other, while the points from neighboring droplet transitions are far apart, as shown in Figure 7. Based on this feature, we propose a feature extraction method for the droplet transition based on DBSCAN to extract the signals corresponding to one droplet transition to count the number of droplet transitions in one sampling period. DBSCAN is a typical algorithm used for analyzing data structures based on density; it can obtain arbitrarily shaped clusters and not have to specify the number of classes [37]. DBSCAN classifies the transfer signal points into *c* clusters and one noise cluster, where *c* represents the number of droplet transitions $N_{occur}=c$. The feature signal of the droplet transition, $D_{i}$, is obtained by Equation (13), and the extraction result is shown in Figure 8.

(13) $$D_{i}=C_{i},1\leq i\leq c$$

where $D_{i}$ is the *i*th droplet transition, and $C_{i}$ is the *i*th cluster.

### 2.4. Real-Time Extraction for Droplet Transfer Frequency and Droplet Transition Uniformity

If we find *c* droplet transitions during time *T* in one sampling period, the droplet transfer frequency is calculated by Equation (14):

(14) f=c/T

During GTAW—with uniform welding speed and wire feed speed—the uniformity of the interval time between neighboring droplet transitions reflects the uniformity of the welding bead size, so it can be used as feedback to control the welding bead. Based on this principle, we propose a method for calculating droplet transition uniformity using arc sensing, which uses the variance of the interval time of neighboring droplet transitions. The steps are as follows:

Assuming that there are *c* droplet transitions during time *T* in one sampling period, $V_{c}^{i}$ is the voltage data point set of the *i*th droplet transition, $N_{c}^{i}$ is the amount of data in this set, $T_{ci}^{k}$ is the time corresponding to the *k*th point in the set.

First, we calculate the barycenter of the *i*th droplet transition using the dataset, $V_{c}^{i}$, with Equation (15):

(15) $$\bar{T_{i}}=\sum T_{ci}^{k}/N_{c}^{i}$$

where $\bar{T_{i}}$ is the barycenter of the *i*th droplet transition

Second, we calculate the distance between the neighboring barycenter with Equation (16):

(16) $$D_{Ti}=\bar{T_{i+1}}-\bar{T_{i}}$$

Third, we calculate the average of all the distance values between the neighboring barycenter with Equation (17):

(17) $$\bar{D_{Ti}}=\sum D_{Ti}/\left(C-1\right)$$

Fourth, we calculate the variance of the distance with the Equation (18):

(18) $$T_{s^{2}}=\sum {\left(D_{Ti}-\bar{D_{Ti}}\right)}^{2}$$

If $T_{s^{2}}$ is relatively high, droplet transition uniformity is relatively bad, if $T_{s^{2}}$ is relatively low, droplet transition uniformity is relatively good.

### 2.5. Algorithm Efficiency Analysis

The real-time monitoring method for droplet transfer frequency in wire-filled GTAW using arc sensing consists of four main algorithms. To verify the efficiency of the algorithm, we processed 50 sets of data—where there were 1000 sampling data pieces in every set—to test the algorithm’s efficiency and calculate the average running time of all the algorithms using MATLAB R2022a in IPC (industrial personal computer).

For one dataset, the average running times of all four algorithms are shown in Table 1. This method meets the needs of real-time monitoring as the total computational time for all algorithms combined is only 27.86 ms.

## 3. Experimental Results and Analysis

### 3.1. Experimental Equipment

**Figure 9:**
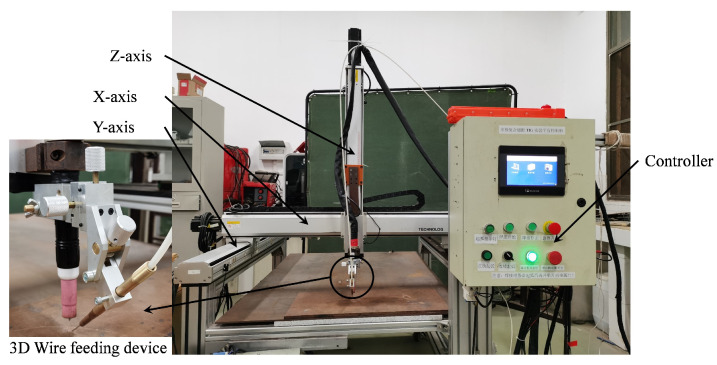
The experiment platform.

We built a welding robot experimental platform, as shown in Figure 9, to conduct a performance verification on the real-time monitoring method for droplet transfer frequency in wire-filled GTAW using arc sensing. The welding robot experimental platform consists of a controller, an actuator (operating range: 1600 mm × 1600 mm × 1600 mm), a power supply (YC–300 BP), and an automatic wire feeding device (YJ–105), etc. There are three degrees in the robot. The welding torch can move left or right along the *X-axis* and move up or down along the *Z-axis*, and the welding torch can weld along the *Y-axis*. In addition, we used a high-speed camera system (AcutEye, Ketianjian Company, China) and a hall sensor (CHV-50VD, Beijing Sensor Electronics Company, Beijing, China).

### 3.2. Real-Time Monitoring Experiment of Droplet Transfer Frequency

**Figure 10:**
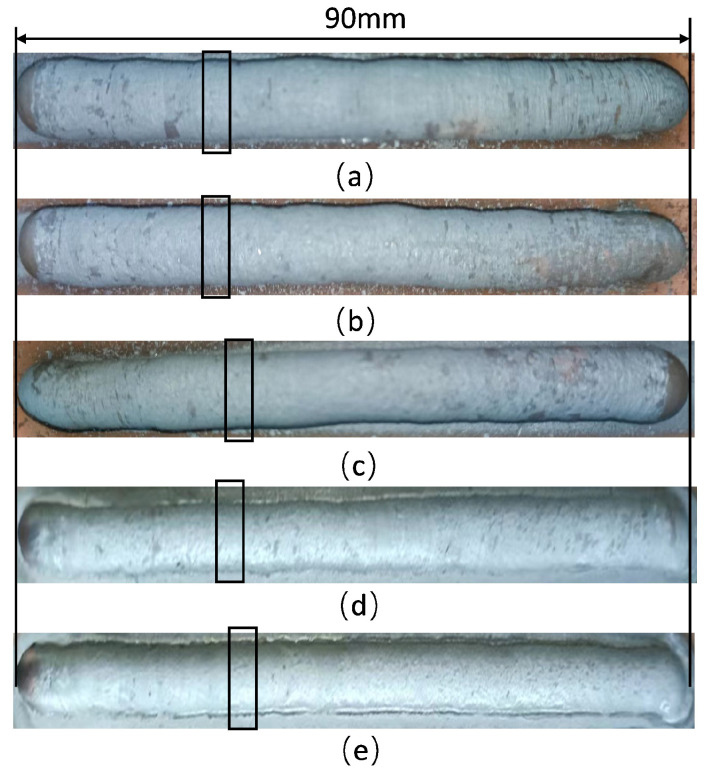
The welding beads: (**a**) welding speed-50 mm/min, wire feeding speed-1200 mm/min, welding current-80 A; (**b**) welding speed-60 mm/min, wire feeding speed-1500 mm/min, welding current-100 A; (**c**) welding speed-70 mm/min, wire feeding speed-1800 mm/min, welding current-120 A; (**d**) welding speed-90 mm/min, wire feeding speed-2400 mm/min, welding current-140 A; (**e**) welding speed-110 mm/min, wire feeding speed-2700 mm/min, welding current-160 A.

We conducted experiments using different typical parameters on the real-time monitoring method of droplet transition frequency. The wire was made of 304 steel with a diameter of 1.2 mm. The diameter of the tungsten electrode was 2.4 mm, and the shielding gas was 99.99% argon. The arc length was 14 mm, and the wire-filled height (the vertical distance between the top of the wire and the workpiece) was 6 mm. The other parameters are shown in Table 2. The weld beads corresponding to the parameters are shown in Figure 10.

**Figure 11:**
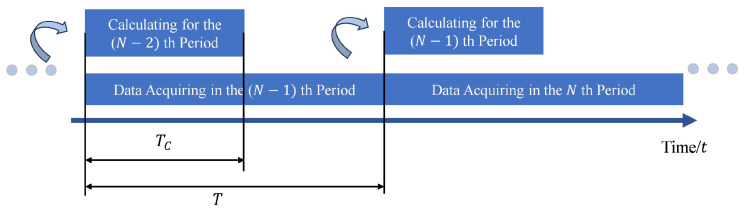
Timing diagram of the real-time monitoring of the droplet transition in wire-filled GTAW.

During GTAW, this method acquires the arc voltage signal and simultaneously extracts the information about droplet transition. The timing diagram is shown in Figure 11, where *T* is the sampling period for the arc signal and $T_{c}$ denotes the period of extracting droplet transfer information.

The initiation of a high frequency and high voltage arc, along with electromagnetic interference, will affect the controller. To avoid these effects, we isolate the data acquisition card from the arc voltage using a linear voltage sensor. The IPC conducts A/D sampling for a period of 2 s (sampling period *T* = 2 s), and the sampling frequency is 500 Hz.

**Figure 12:**
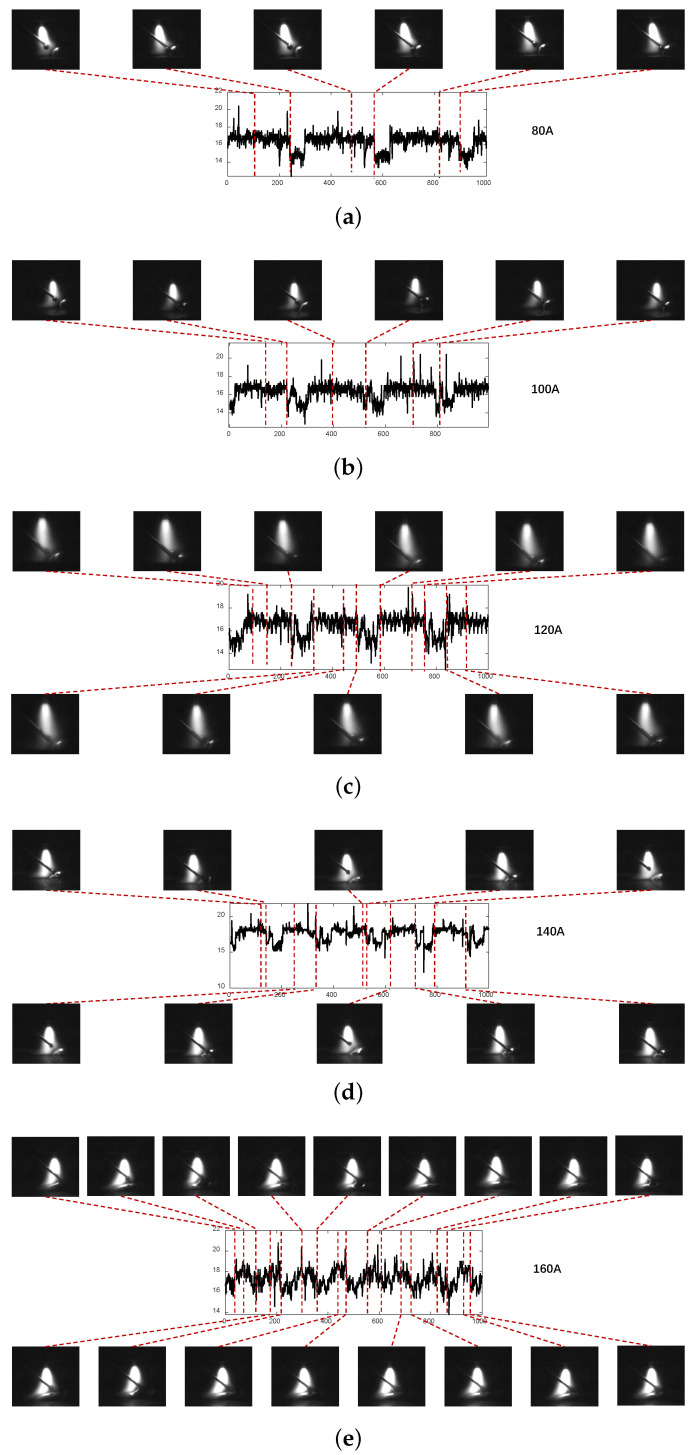
The original signal and the corresponding arc photos at different welding currents: (**a**) welding current-80 A; (**b**) welding current-100 A; (**c**) welding current-120 A; (**d**) welding current-140 A; (**e**) welding current-160 A.

**Figure 13:**
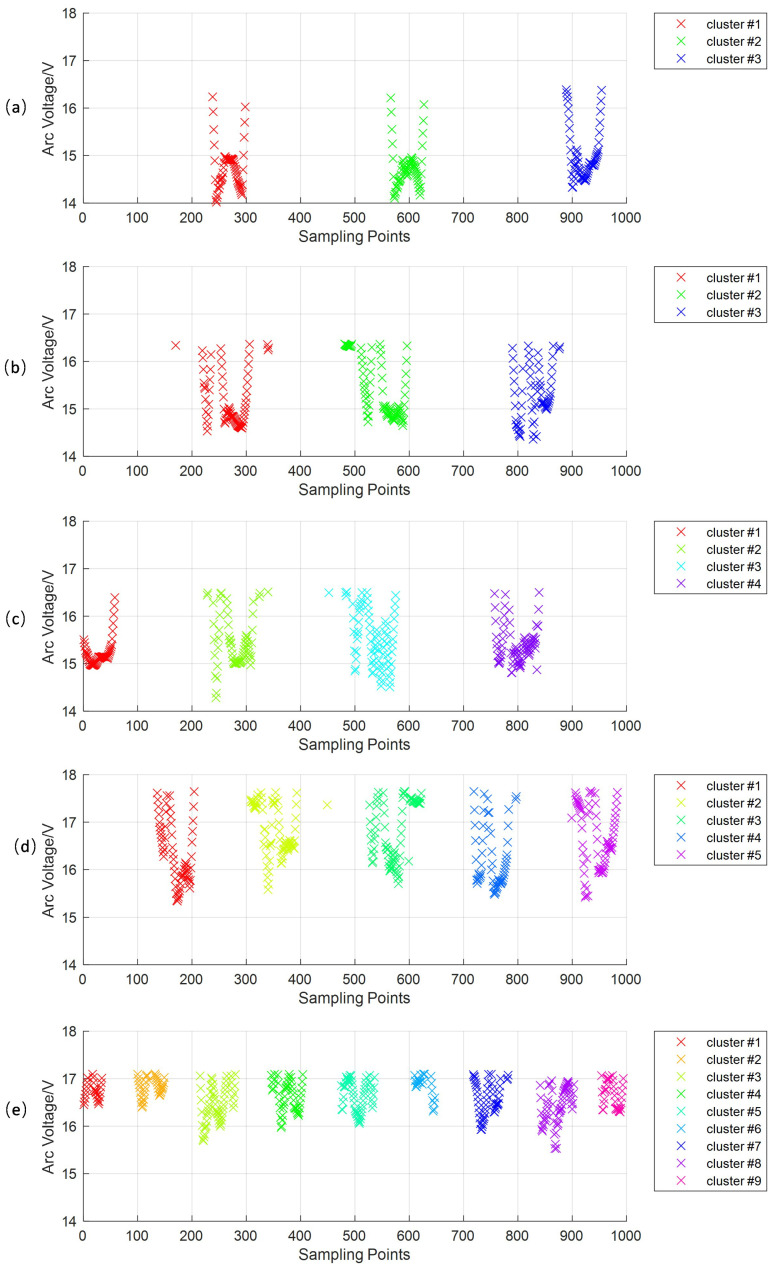
The extraction results of the feature signals of the droplet transition: (**a**) welding current-80 A; (**b**) welding current-100 A; (**c**) welding current-120 A; (**d**) welding current-140 A; (**e**) welding current-160 A.

The original arc voltage signal is shown in Figure 12. It indicates that there will be much noise and a decline of about 2V in every droplet transition. To make the real-time monitoring of the droplet transition accurate, we propose a filtering method based on wavelet transform to filter out noise. The filtered arc voltage signal retains the changing trends of the original one without too much high-frequency noise. Then, we segment the droplet transfer signal and droplet growth signal into two datasets using the arc signal segmentation method based on the OTSU algorithm. For the droplet transfer signal dataset, we extract the feature signal of every droplet transition, and the results are shown in Figure 13.

In Figure 13, the signal points corresponding to the same droplet transition are gathered into one cluster. The number of clusters corresponds to the number of droplet transitions in one sampling period. We can calculate the droplet transfer frequency using Equation (14), and the results are shown in Table 3. In Table 3, the droplet transition frequency changes from 1.5 Hz to 4.50 Hz when the arc current changes from 80 A to 160 A. The real-time monitoring method of the droplet transition we propose can be applied to different conditions that use different welding parameters.

For more information, we propose a calculation method for droplet transition uniformity. This method uses the distance variance between neighboring droplet transitions to represent droplet transition uniformity, $T_{s^{2}}$. As the value of $T_{s^{2}}$ declines, droplet transition uniformity becomes better. The results shown in Table 3 suggest that $T_{s^{2}}<0.4$, which is a small value, suggesting that the uniformity of the weld beads, as shown in Figure 10, is great.

### 3.3. Error Analysis

To examine the accuracy of the real-time monitoring method, we chose 10 positions in every bead in experiments (a) to (e), shot with a high-speed camera to observe the droplet transition. Shooting in every position lasts 2 s, which is equal to the sampling period. The real droplet transfer frequency can be observed from the photos shot by a high-speed camera. Then, the droplet transfer frequency from the proposed monitoring method is compared to the real droplet transfer frequency observed from photos so that the monitoring error of the monitoring method for droplet transfer frequency using arc sensing can be obtained by Equation (19). As shown in Table 4, the monitoring error of the method is less than 0.05 Hz.

(19) $$E=\sum_{i=1}^{i=10}\left|\right|f_{si}-f_{ci}\left|\right|/10$$

where $f_{si}$ is the droplet transfer frequency obtained by photos in the *i*th position; $f_{ci}$ is the droplet transfer frequency of the *i*th position obtained by the real-time monitoring method; and *E* is the average monitoring error.

### 3.4. Experiments on the Relationship between the Uniformity of Droplet Transitions and the Uniformity of Weld Width

**Figure 14:**
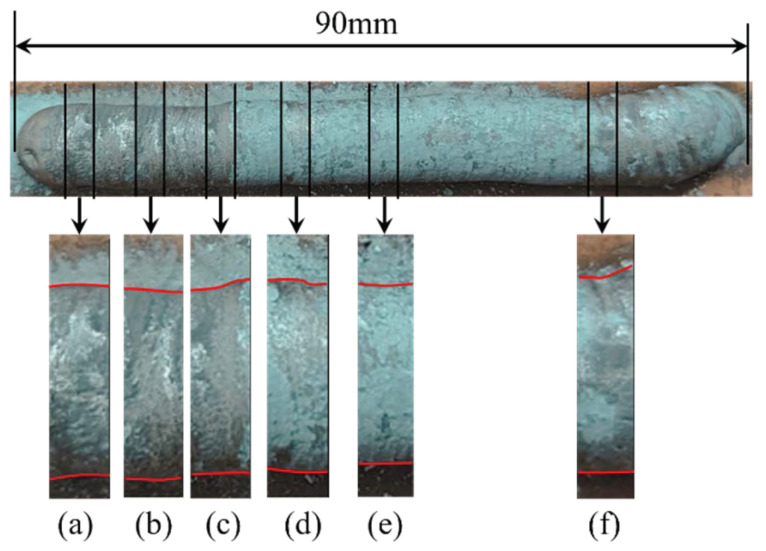
The weld bead welded on an unfixed workpiece: (**a**) the first selected segment; (**b**) the second selected segment; (**c**) the third selected segment; (**d**) the fourth selected segment; (**e**) the fifth selected segment; (**f**) the sixth selected segment.

Changes in arc length, due to workpiece deformation and tungsten electrode wear, along with variations in wire feeding position caused by wire bending, will change the droplet transfer frequency and droplet transition uniformity, leading to droplet transfer frequency changes in real time. To explore the relationship between the uniformity of the bead appearance and the uniformity of the droplet transition, we conducted an experiment on an unfixed workpiece. The parameters are shown in Table 5 and the corresponding weld bead is shown in Figure 14.

**Figure 15:**
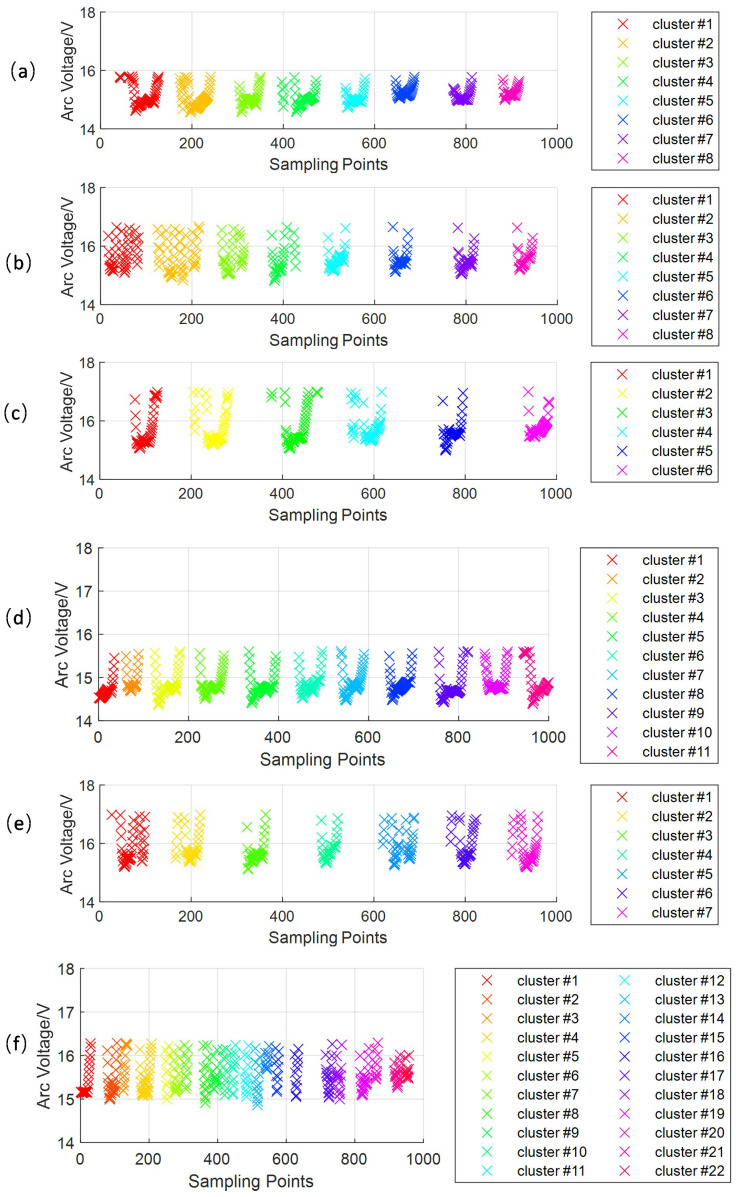
The droplet transition extraction results of the feature signals of the droplet transition: (**a**) the extraction resut of the first selected segment; (**b**) the extraction resut of the second selected segment; (**c**) the extraction resut of the third selected segment; (**d**) the extraction resut of the fourth selected segment; (**e**) the extraction resut of the fifth selected segment; (**f**) the extraction resut of the sixth selected segment.

Figure 14 demonstrates that workpiece deformation can lead to the non-uniformity of weld beads during GTAW. We chose six different segments to process the signals while welding. The corresponding weld bead segments (a) to (f) are shown in Figure 14, and the feature extraction result is shown in Figure 15. The droplet transition frequency and droplet transition uniformity are demonstrated in Table 6. The droplet transition frequency changes from 3.0 Hz to 11.0 Hz, caused by workpiece deformation, and the $T_{s^{2}}$ changes from 0.0715 to 0.7894, so there is no necessary connection between them. As shown in Figure 14, the bead width uniformity of samples (a), (b), and (e) is relatively great, and the corresponding $T_{s^{2}}$ values are 0.0715, 0.2670, and 0.2467. These values are relatively lower. But the weld bead uniformity of samples (c), (d), and (f) is relatively worse, and the corresponding $T_{s^{2}}$ values are 0.5031, 0.5391, and 0.7894. These values are relatively higher. We can conclude that with $T_{s^{2}}$ increasing, which means that the uniformity of the droplet transition becomes worse, the weld bead width uniformity worsens. This principle can be used to monitor the weld bead width uniformity.

## 4. Conclusions

In this paper, we propose a real-time monitoring method for droplet transfer frequency in wire-filled GTAW using arc sensing and establish a real-time monitoring system for droplet transfer frequency, achieving real-time monitoring of the droplet transfer frequency in wire-filled GTAW. The conclusions are as follows:

1. A real-time monitoring method for droplet transfer frequency using arc sensing is proposed. In this method, the original signal acquired by a hall sensor is filtered by the wavelet transform method; the feature signal of the droplet transition is extracted by the arc signal segmentation method based on OTSU, and the feature extraction method for droplet transition based on DBSCAN is proposed. The real-time monitoring method we propose satisfies the demand for the real-time monitoring of droplet transfer frequency since the running time of the four main algorithms is 27.86 ms in total.
2. We propose a concept of droplet transition uniformity. The experiments prove that weld width uniformity and droplet transition uniformity are positively correlated under conditions of uniform wire feeding and welding speeds. This principle can be used to monitor the weld bead width uniformity.
3. The results of the experiments with typical parameters show that the maximal monitoring error is 0.05 Hz. This method holds promise for widespread use in monitoring and providing feedback control for droplet transfer in surface repairing and WAAM based on GTAW.

## Figures and Tables

**Table 1:** The processing times of the four main algorithms.

Algorithm	Running Time/ms
A filtering method based on wavelet transform	3.12
An arc signal segmentation method based on OTSU	6.80
A feature extraction method for droplet transition based on DBSCAN	16.50
Calculating for droplet transfer frequency and the uniformity of transition	1.44
Total	27.86

**Table 2:** Welding parameters.

Number	Welding Speed (mm/min)	Wire Feeding Speed (mm/min)	Welding Current (A)
(a)	50	1200	80
(b)	60	1500	100
(c)	70	1800	120
(d)	90	2400	140
(e)	110	2700	160

**Table 3:** The results of droplet transfer frequency and droplet transition uniformity.

Number	Droplet Transfer Frequency (f/Hz)	The Uniformity of Droplet Transitions ($T_{s^{2}}$)
(a)	1.50	0.0153
(b)	1.50	0.0098
(c)	2.00	0.1080
(d)	2.50	0.3879
(e)	4.50	0.1788

**Table 4:** Average error.

Number	(a)	(b)	(c)	(d)	(e)
*E* (Hz)	0.02	0.01	0.01	0.05	0.05

**Table 5:** Welding parameters.

Welding Speed (mm/min)	Wire Feeding Speed (mm/min)	Welding Current (A)
60	1500	100

**Table 6:** The calculating results of droplet transfer frequency and droplet transition uniformity.

Number	Droplet Transfer Frequency f (Hz)	The Uniformity of Droplet Transitions $T_{s^{2}}$
(a)	4.0	0.0715
(b)	4.0	0.2670
(c)	3.0	0.5031
(d)	5.5	0.5391
(e)	3.5	0.2467
(f)	11.0	0.7894

## Data Availability

Data are contained within the article.
